# How Successful Is AI in Developing Postsurgical Wound Care Education Material?

**DOI:** 10.1111/wrr.70041

**Published:** 2025-05-13

**Authors:** Yeliz Sürme, Handan Topan, Gülseren Maraş Baydoğan

**Affiliations:** ^1^ Faculty of Health Sciences, Surgical Nursing Erciyes University Kayseri Türkiye

**Keywords:** artificial intelligence, patient education, patient education material, surgery, wound care

## Abstract

ChatGPT can be used as an aid in education, research and clinical management. This study was conducted using the ChatGPT 4.0 program to develop artificial intelligence‐supported wound care education material that can be read and understood by patients discharged after surgery. In this methodological study, while creating wound care education material, the education needs of the patients were determined first. Then, the education content was created in the ChatGPT 4 program. Expert opinion was taken for the clarity, applicability, accuracy and quality of the education content. The Turkish readability index of the education material was found to be 68.9 and easily understandable. The Automated Readability Index was found to be 9.29, the Simple Measure of Gobbledygook 7.89, the Flesch‐Kincaid 8.07, the Flesch Reading Ease 59.0 and the Average Reading Level Consensus 9.99, which are frequently used in health literature. The PEMAT understandability and applicability score averages were determined 93.90 ± 6.11 (84–100) and 90.20 ± 8.66, respectively. The Global Quality Scale score average was found to be 4.40 ± 0.69. This study reveals that ChatGPT provides understandable, applicable, accurate and high‐quality postoperative wound care education material.

## 1 Introduction

Worldwide, an estimated 4511 operations are performed per 100,000 people each year, with 1 in 22 people undergoing surgery each year. Surgical wounds are the most common wounds in acute care settings and are associated with a variety of complications, including bleeding and wound dehiscence. Surgical site infections are the most common and most preventable hospital‐acquired infections [1, 2].

One in four patients may develop postoperative complications within 14 days after discharge. It is stated that surgical wound complications constitute almost 4% of total healthcare system costs and this rate is increasing [1].

Some factors affect wound healing after surgery. These factors include intrinsic factors such as advanced age, malnutrition, metabolic diseases, smoking, obesity, hypoxia and length of preoperative period and extrinsic factors such as preoperative skin preparation and skin antiseptics, antibiotic prophylaxis, inadequate sterilisation of surgical instruments, surgical drains, surgical hand scrubs and dressing techniques [2]. The most critical factor in the development of surgical wound complications is not the lack of evidence‐based guidelines, but rather knowing to implement these guidelines, adopting the right attitude, demonstrating intent and effectively managing care in this regard. Wound management in critically ill patients is a vital component of critical nursing care, and healthcare practitioners should pay close attention to wound care [3].

It has been stated that half of the repeated hospitalizations related to wound complications could be prevented with postoperative education and closer follow‐up [4]. Given the widespread use of surgical procedures and the potential burden of wound complications such as surgical wound dehiscence and infection for patients and their families, it is inevitable that patients will seek information about surgical wound care [5]. A review stated that patients often lack knowledge about the impact of surgery on their ability to return to normal daily living activities, how to identify complications that may develop and how to respond. Patients also reported a lack of information about the early stages of recovery at hospital discharge [6]. In a meta‐ethnography study, all participants stated that they needed more information about surgical treatment and the recovery process [7].

In addition to verbal education to meet the patient's information needs, personalised patient education materials have been reported to improve patient satisfaction and health literacy, leading to improved patient care [8].

Due to the significant improvement in health literacy, artificial intelligence (AI) has been increasingly used in particularly in the field of natural language processing [9]. ChatGPT, a chatbot‐based technology, is a type of software that produces human‐like conversational texts [10]. ChatGPT has not only been accepted as an author in some journals [11], but it has been stated that it can be used as an aid in education, research and clinical management [10]. Effectively prepared educational materials can reduce patients' anxiety and increase their compliance with explanations, while also helping them understand medical complications. Educational materials are considered effective if they contain readable, understandable and memorable information [12].

Therefore, this study was conducted using the ChatGPT 4.0 program to develop an AI‐supported wound care education material that patients who will be discharged after surgery can read and understand.

### 1.1 Research Questions

- How understandable is the wound care training material developed for post‐surgical wound care for patients?
- How applicable is the wound care training material developed for post‐surgical wound care for patients?
- How readable is the wound care training material developed for post‐surgical wound care in Turkish?
- What is the quality score of the wound care training material developed for post‐surgical wound care?

## 2 Methods

### 2.1 Design

In this study, which was conducted to develop an AI‐supported wound care education material, a methodological design was used. STROBE (strengthening the reporting of observational studies in epidemiology) checklist guidelines were followed throughout the study (Supporting Information).

### 2.2 Settings

While creating the wound care education material, the education needs of the patients were first determined. Then, the education content was created in the ChatGPT 4 program.

OpenAI released ChatGPT version 4.0 on 14 March 2023. This new version introduced significant improvements such as understanding more nuanced prompts and more context‐aware responses. This version also offers a paid component that requires users to subscribe for $20 per month to access some advanced features [13].

The readability of the draft material was determined, and expert opinion was obtained regarding the educational content created.

### 2.3 Determining the Educational Needs of Patients

The researchers' experience and literature review were used in determining the study topic. Researchers, who are both academicians with clinical experience in surgical services, observed that surgical patients needed training on wound care after surgery. Researchers' literature review also verified patients' lack of knowledge on this issue [6, 7, 14].

### 2.4 Creating Educational Content in ChapGPT

Researchers have scanned the literature on postoperative wound care [1, 15, 16, 17, 18] and reached systematic reviews, meta‐analyses and protocols. The references accessed were added to the ChatGPT 4.0 program and a command was given to create educational content under the titles specified in Table 1 in line with these references. The AI‐supported Dall‐E program was used to create visuals in the wound care educational material. DALL‐E is a revolutionary AI tool that can generate images from text‐based descriptions. Developed by OpenAI, this tool uses advanced deep‐learning models to produce high‐quality, detailed images [19].

**TABLE 1:** Prompts given for wound care education material.

Prompts
I am a wound care nurse in a surgical ward. Could you create educational material on wound healing for the patient who will be discharged with a postoperative wound in a format understandable to the patient?
2 I am a wound care nurse in a surgical ward. Could you create educational material about what to do to prevent wound infection for the patient who will be discharged with a wound after surgery, in a format understandable to the patient?
3 I am a wound care nurse in a surgical ward. Could you create educational material on wound care for the patient who will be discharged with a postoperative wound in a format understandable to the patient?
4 I am a wound care nurse in a surgical ward. Could you create educational material about baths for the patient who will be discharged with a postoperative wound in a format understandable to the patient?
5 I am a wound care nurse in a surgical ward. Could you create educational material about nutrition for the patients who will be discharged with a postoperative wound in a way that the patient understands?
6 I am a wound care nurse in a surgical ward. Could you create educational material outlining potential wound complications, their symptoms and necessary actions for a patient being discharged with a postoperative wound in a format understandable to the patient?

**FIGURE 1:**
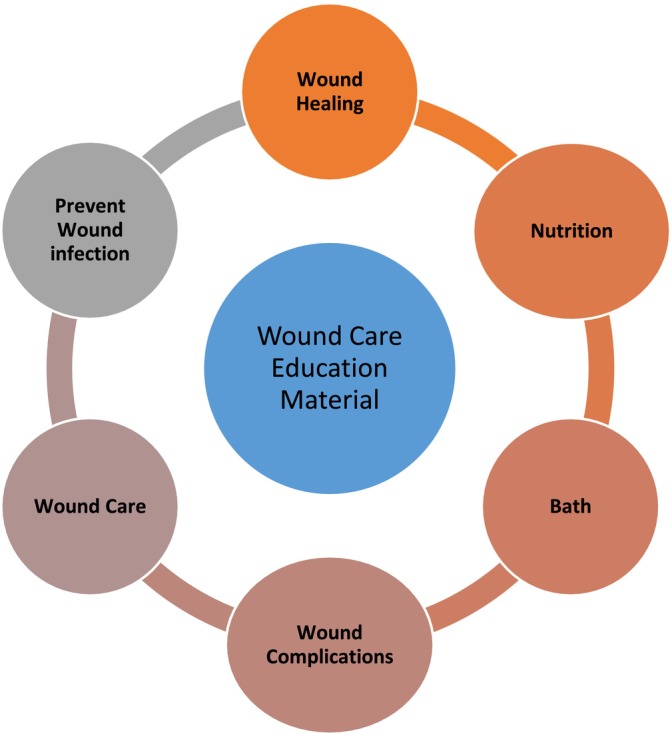
Contents of wound care education material.

The wound care educational material consisted of 23 pages and 2803 words. Contents of wound care education material are presented in Figure 1.

### 2.5 Assessing Readability

The concept of readability is a term generally used in the evaluation of printed educational materials. In other words, it is whether a text created in a language can be followed easily by readers. The readability formula for Turkish was developed by Ateşman by adapting the Flesch formula to Turkish. According to Ateşman, ‘The average sentence length in Turkish is 9‐10 words, and the average word length is 2–6 syllables’. According to the formula, the readability level is between 0 and 100. As the obtained score approaches 100, the readability of the text becomes easier, and as it approaches 0, the readability of the text becomes more difficult [20] (http://okunabilirlikindeksi.com).

Readability was also evaluated using the ‘Simple Measure of Gobbledygook (SMOG)’, ‘Flesch‐Kincaid’ and ‘Flesch Reading Ease’ formulas, which are frequently used in the health literature as stated by Wang et al. [21]. Finally, it was assessed using the ‘Automatic Readability Index’ formula developed by Smith and Senter [22] (https://readabilityformulas.com).

### 2.6 Obtaining Expert Opinions on the Educational Material

The validity of the AI‐supported wound care education material was presented to the opinion of 10 experts who have doctoral degrees in surgical nursing and study in the field of wound care. The experts evaluated the understandability and applicability of the educational material using the Patient Education Materials Assessment Tool (PEMAT‐P) and the quality of the educational material using the Global Quality Scale.

### 2.7 Patient Education Materials Assessment Tool (PEMAT)

The PEMAT was developed by Shoemaker et al. in 2014 to evaluate and compare the understandability and applicability of printable and audiovisual educational materials [23], and its Turkish validity and reliability study was conducted by Akkoç and Orkun 2023 [24]. Understandability is achieved when individuals with different levels of education and health literacy can understand and explain the basic messages given. Applicability occurs when individuals can determine which steps to take based on the information presented to them. PEMAT has two versions: Printable materials—patient education material evaluation tool (PEMAT‐P) and Audiovisual materials—patient education material evaluation tool (PEMAT‐A/V). In this study, the ‘Printable materials patient education material evaluation tool (PEMAT‐P)’ was used to evaluate the understandability and applicability of AI‐supported wound care education material for patients who will be discharged after surgery.

Printable materials consist of a total of 24 items, 17 of which evaluate understandability and 7 items evaluate applicability for the patient education material evaluation tool (PEMAT‐P). The scale is scored as ‘0’ (*disagree*), ‘1’ (*agree*) and ‘*not applicable*’. Scoring is obtained by dividing the total score by the possible score and multiplying by 100. The final score is evaluated between 0 and 100 in terms of understandability and applicability. The higher the score, the higher the understandability or applicability of the material. Cronbach's alpha reliability coefficient for PEMAT‐P has been reported as 0.901 [24]. In our study, Cronbach's alpha reliability coefficient was found to be 0.79.

### 2.8 Quality Score of the Educational Materials (GQS)

The appropriateness and quality of the content of the wound care educational material were assessed using the Global Quality Scale. The Global Quality Scale was developed by Bernard et al. [25]. It is rated using a five‐point Likert scale. The scores refer to the quality of the educational material and the extent to which the evaluator finds it useful for patients. Accordingly, a score of 1 indicates poor quality and a score of 5 indicates excellent quality. (Table 2) [26, 27].

**TABLE 2:** GQS criteria.

Criteria	Scores
Poor quality, poor flow of the site, most information missing, not at all useful for patients	1
Generally poor quality and poor flow, some information listed but many important topics missing, of very limited use to patients	2
Moderate quality, suboptimal flow, some important information is adequately discussed but others poorly discussed, somewhat useful for patients	3
Good quality and generally good flow, most of the relevant information is listed, but some topics not covered, useful for patients	4
Excellent quality and excellent flow, very useful for patients	5

### 2.9 Statistical Analysis

Statistical analyses were performed using SPSS 25.0 (Statistical Package for Social Science). Descriptive statistics were presented as unit count (*n*), percentage (%) and mean ± standard deviation (*X̄* ± SD). Intra‐class Correlation Coefficient analysis was used to calculate the experts' internal consistency. A *p*‐value of *p* < 0.05 was considered statistically significant for all results.

### 2.10 Ethics Statement

In this study, ethical approval was not obtained as it involved the development of wound care educational materials and did not include patient participants.

## 3 Results

### 3.1 Readability of the Education Material

The developed wound care education material was found to have a Turkish readability index of 68.9 and was easily understandable. Frequently used in health literature, the Automated Readability Index (ARI) was found to be 9.29 (slightly difficult), the Simple Measure of Gobbledygook (SMOG) was found to be 7.89 (average—slightly difficult), Flesch‐Kincaid was found to be 8.07 (average—slightly difficult), Flesch Reading Ease was found to be 59.0 (fairly difficult), and Average Reading Level Consensus was found to be 9.99 (somewhat difficult) (Table 3).

**TABLE 3:** Readability index and levels of wound care education material.

	Readability index	Readability level
Ateşman	68.9	9th or 10th grade students (easily understandable)
Automated Readability Index (ARI)	9.26	10th grade slightly difficult
Simple Measure of Gobbledygook (SMOG)	7.89	8th grade Average—slightly difficult
Flesch‐Kincaid	8.07	8th grade Average—slightly difficult
Flesch Reading Ease	59.0	10th to 12th grade Fairly difficult
Average Reading Level Consensus	9.99	10th grade Somewhat difficult

### 3.2 Inter‐Rater Reliability

The internal consistency coefficient between experts was found to be 0.79 (95% CI [0.187–0.950], *p* < 0.05).

In most of the PEMAT items (15 items), 90% and above of the experts responded that they agreed.

### 3.3 Understandability and Applicability of the Education Material

The PEMAT understandability and applicability score averages were determined as 93.90 ± 6.11 (84–100) and 90.20 ± 8.66, respectively (Table 4).

**TABLE 4:** Experts' responses to PEMAT items and PEMAT and GQS Criteria averages for wound care education material.

PEMAT items	Agree *n* (%)	Disagree *n* (%)	Not applicable (%)
**Understandability**			
The material fully explains its purpose.	10 (100.0)		
2 The material does not contain any information or meaning that would distract from its purpose.	9 (90.0)	1 (10.0)	
3 The material uses everyday, common language.	10 (100.0)		
4 Medical terms are used only to familiarise the reader/listener with the terms. When used, medical terms are defined.	10 (100.0)		
5 The material uses an active verb.	10 (100.0)		
6 The numbers appearing in the material are clear and easy to understand.	8 (80.0)	1 (10.0)	1 (10.0)
7 The user is not expected to make calculations in the material.	10 (100.0)		
8 Information in the material is divided into short sections or chunks.	10 (100.0)		
9 The sections of the material have informative headings.	10 (100.0)		
10 The material presents information in a logical order.	10 (100.0)		
11 The material includes a summary.	10 (100.0)		
12 The material uses visual cues (e.g., arrows, boxes, bullets, bold, larger font, highlighting) to draw attention to key points.	9 (90.0)	1 (10.0)	
13 The material uses visual aids to make the content easier to understand (e.g., representation of healthy portion sizes).	6 (60.0)	4 (40.0)	
14 Visual aids in the material support understanding rather than distract from the content.	4 (40.0)		6 (60.0)
15 The visual aids of the material have clear titles or subtitles.	3 (10.0)		7 (70.0)
16 The material uses neat and clear drawings and photographs.	3 (10.0)		7 (70.0)
17 The material uses simple tables with concise and clear row and column headings.	1 (10.0)		9 (90.0)
**Applicability**			
18 The material clearly describes at least one action that the user can take.	10 (100.0)		
19 The material addresses the user directly when describing actions.	10 (100.0)		
20 The material breaks down any action into manageable, clear steps.	10 (100.0)		
21 The material provides a concrete tool (e.g., planners, checklists) that can help the user take action.	9 (90.0)	1 (10.0)	
22 The material provides simple instructions or examples of how to do calculations.	7 (70.0)		3 (30.0)
23 The material explains how to use charts, graphs, tables and diagrams for action.		1 (10.0)	9 (90.0)
24 The material uses visual aids to facilitate following instructions.	6 (60.0)	4 (40.0)	

	Mean ± SD	Min	Max
PEMAT understandability (0–100)	93.90 ± 6.11	84	100
PEMAT applicability (0–100)	90.20 ± 8.66	80	100
GQS criteria (1–5)	4.40 ± 0.69	3	5

### 3.4 Quality Score of the Education Material

The Global Quality Scale score average, which evaluates the appropriateness and quality of the content of the wound care educational material, was found to be 4.40 ± 0.69 (3–5) (Table 4).

## 4 Discussion

In a study, it was stated that 11.5% of patients experienced wound complications such as wound dehiscence and infection after surgery, and 1.9% of these patients underwent reoperation for the treatment of wound complications. Preoperative counselling and postoperative wound management should be provided to minimise the risk of surgical site infection and prevent wound problems [28]. The rapid development of AI may be an innovative method that will help reduce the burden faced by patients and healthcare providers in the field of wound care [29]. In this study, an AI‐supported wound care educational material was created to be used in patient education aimed at preventing post‐surgical wound complications.

In today's world where digital technologies, especially AI, are widely used, patients are seeking information about their diseases and treatment options using internet‐based applications. Although there is a significant amount of information on the internet, patients' use of this information depends on the readability and understandability of the information [30]. In this study, the Turkish readability index of the AI‐supported wound care education material was 68.9, and it was easily understandable. Frequently used in health literature, the Automated Readability Index (ARI) was 9.29 (slightly difficult), the Simple Measure of Gobbledygook (SMOG) was 7.89 (average—slightly difficult, 8th grade), the Flesch‐Kincaid was 8.07 (average—slightly difficult, 8th grade), the Flesch Reading Ease was 59.0 (fairly difficult, 10th to 12th grade) and the Average Reading Level Consensus was 9.99 (somewhat difficult, 10th grade). The Readability Index parameters can be said to be slightly difficult in general. Similar to our study, in a study conducted to evaluate the readability, quality and reliability of online patient education materials regarding Transcutaneous electrical nerve stimulation (TENS), the Flesch Reading Ease Score was 47.91 (difficult), the mean Flesch‐Kincaid Grade Level and Simple Measure of Gobbledygook were 11.20 ± 2.85 and 10.53 ± 2.11 difficult, respectively. In a study evaluating the readability of patient education material created by ChatGPT 4.0 ChatBot on ophthalmology, it was reported that non‐prompted materials had the highest readability scores in all readability indices and may be the most difficult material to read in this form (the Flesch Reading Ease Score: 36.5; the Simple Measure of Gobbledygook: 14.7). In the same study, when a command was given to output patient education material at a 6th‐grade reading level, ChatGPT 4.0 was able to reduce the average word count from 683.3 to 719.6 words and also improve reading indices (the Flesch Reading Ease Score: 67.9; the Simple Measure of Gobbledygook: 10.2). These findings suggest that the material generated by the AI chatbot can be most easily understood with additional command‐based guidance [31]. Furthermore, it is noted that enhancing the readability of the materials through visual aids can be beneficial [32]. In our study, the ease of understanding the material in Turkish demonstrates its usability by patients.

The PEMAT‐P understandability and applicability score averages, which were assessed by 10 academicians who are experts in their fields, were found to be 93.90 ± 6.11 and 90.20 ± 8.66, respectively throughout the study. In most of the PEMAT items (15 items), 90% and above of the experts responded with ‘agree’. There are various results in the literature on the evaluation of AI‐supported educational materials with PEMAT. In a study aimed at evaluating the performance of three conversational agents (ChatGPT, Bard and Copilot) and a reliable website in responding to real patient questions about strabismus, ChatGPT's PEMAT‐U and PEMAT‐A scores were found to be 67.8 and 61.1, respectively [33]. A comparison of educational materials created by ChatGPT and Google Bard was made on three subheadings on obstructive sleep apnea. The PEMAT‐U score of the material created by ChatGPT using the chatbot ranged between 89.94 and 90.86; PEMAT‐A between 72.22 and 77.14 and was found to be significantly higher [34] Our study revealed that the understandability and applicability scores of the wound care educational material were relatively higher compared to the literature. This may be attributed to the material being prepared not as a conversational format but rather as an educational booklet supported with visuals and designed based on prompts to include certain references in its preparation.

The internal consistency coefficient between experts for the prepared wound care education material was found to be 0.79 (95% CI [0.187–0.950], *p* < 0.05). Opinions of two experts were obtained for the prepared material on obstructive sleep apnea and the correlation between the raters was reported as 0.957 (95% CI 0.943: 0.968) [34]. In another study, the reliability between the two raters was reported as 0.87. It is widely accepted that an ICC between 0.75 and 0.9 indicates good reliability, and an ICC > 0.90 indicates excellent reliability [35]. Therefore, we can say that the reliability between experts in our study is at a good level.

PEMAT does not evaluate the accuracy or comprehensiveness of the content. In addition to readability, understandability and applicability, the reliability and quality of information in the digital environment should also be examined [30]. In our study, the content quality of the wound care education material was evaluated by experts using the Global Quality Scale and received an average score of 4.40 ± 0.69 out of 5. This result shows that the accuracy and content quality of the created educational material are at a good level.

### 4.1 The Strengths and Limitations

The strength of this study is that it is the first study to create an AI‐supported educational material on wound care, an area where post‐surgical wound complications are frequently seen. Another strength is that the understandability, applicability and quality of the created wound care educational material were evaluated using a valid and reliable assessment tool and showed a high internal consistency among experts.

There are also some limitations to this study: the comprehensibility, applicability and quality of the educational materials could not be evaluated by the patients. Another limitation of the study is that although the Turkish readability index is easily understandable at the 9th‐10th grade level, patients below this level may have difficulty understanding the text. It is recommended that future studies evaluate AI‐assisted educational material from the perspective of patients and investigate the impact of AI‐assisted education on patient outcomes.

## 5 Conclusion

This study demonstrated that ChatGPT provides post‐surgical wound care education material that is understandable, applicable, content‐accurate and high‐quality, with relatively difficult readability. AI‐powered applications have the potential to revolutionise post‐surgical patient education and engagement.

These results can be considered as an important step to facilitate and encourage the preparation of patient education materials by clinical and academic nurses. Patient education is an important initiative to prevent post‐surgical complications, and the educational methods developed to provide this education have gained a new dimension thanks to the advancement of technology and AI. This research will ensure that advanced technologies such as AI are integrated into patient education practices and that maximum benefit is obtained from technology.

### 5.1 Relevance of Clinical Practice

The emergence of AI‐enabled technologies has significantly impacted both nursing education and practice. Nurses can take an active role in designing and implementing AI systems to ensure that AI technologies are based on patient‐centered care principles and provide maximum patient benefit. Nursing policy should ensure that guidelines are developed and used to oversee the appropriate use of AI in nursing care and patient monitoring. Policymakers should collaborate with physicians, nurses and technology experts to create a regulatory environment that supports integrating AI systems into patient treatment, care, and education.

## Conflicts of Interest

The authors declare no conflicts of interest.

## Supporting information


**Data S1.** wrr70041‐sup‐0001‐Supinfo.

## Data Availability

The data that support the findings of this study are available from the corresponding author upon reasonable request.
